# Transportation Risk Behaviors Among High School Students — Youth Risk Behavior Survey, United States, 2019

**DOI:** 10.15585/mmwr.su6901a9

**Published:** 2020-08-21

**Authors:** Merissa A. Yellman, Leah Bryan, Erin K. Sauber-Schatz, Nancy Brener

**Affiliations:** ^1^Division of Injury Prevention, National Center for Injury Prevention and Control, CDC; ^2^Division of Adolescent and School Health, National Center for HIV/AIDS, Viral Hepatitis, STD, and TB Prevention, CDC

## Abstract

Motor-vehicle crashes are a leading cause of death and nonfatal injury among U.S. adolescents, resulting in approximately 2,500 deaths and 300,000 nonfatal injuries each year. Risk for motor-vehicle crashes and resulting injuries and deaths varies, depending on such behaviors as seat belt use or impaired or distracted driving. Improved understanding of adolescents’ transportation risk behaviors can guide prevention efforts. Therefore, data from the 2019 Youth Risk Behavior Survey were analyzed to determine prevalence of transportation risk behaviors, including not always wearing a seat belt, riding with a driver who had been drinking alcohol (riding with a drinking driver), driving after drinking alcohol, and texting or e-mailing while driving. Differences by student characteristics (age, sex, race/ethnicity, academic grades in school, and sexual identity) were calculated. Multivariable analyses controlling for student characteristics examined associations between risk behaviors. Approximately 43.1% of U.S. high school students did not always wear a seat belt and 16.7% rode with a drinking driver during the 30 days before the survey. Approximately 59.9% of students had driven a car during the 30 days before the survey. Among students who drove, 5.4% had driven after drinking alcohol and 39.0% had texted or e-mailed while driving. Prevalence of not always wearing a seat belt was higher among students who were younger, black, or had lower grades. Riding with a drinking driver was higher among Hispanic students or students with lower grades. Driving after drinking alcohol was higher among students who were older, male, Hispanic, or had lower grades. Texting while driving was higher among older students or white students. Few differences existed by sexual identity. Multivariable analyses revealed that students engaging in one transportation risk behavior were more likely to engage in other transportation risk behaviors. Traffic safety and public health professionals can use these findings to reduce transportation risk behaviors by selecting, implementing, and contextualizing the most appropriate and effective strategies for specific populations and for the environment.

## Introduction

Motor-vehicle crashes are predictable and preventable. However, in the United States, they remain the second leading cause of death among adolescents and the fourth leading cause of nonfatal injury. During 2018, approximately 2,500 adolescents (persons aged 12–19 years) died in motor-vehicle crashes; of those deaths, >75% were occupants of passenger vehicles (i.e., cars, pickup trucks, vans, or sport utility vehicles) (*1*). Motor-vehicle crashes also resulted in approximately 297,000 nonfatal injuries among adolescents during 2018. Moreover, fatal and nonfatal motor-vehicle–crash injuries among adolescents resulted in approximately $12 billion in medical and work-loss costs during 2018 (https://www.cdc.gov/injury/wisqars).

Passenger-related transportation risk behaviors (e.g., nonuse of seat belts or riding with a driver who had been drinking alcohol) increase the risk for injury or death in a crash or risk for a crash itself. Seat belt use among adolescents and young adults is typically lower than among adults of other age groups (*1*) (https://crashstats.nhtsa.dot.gov/Api/Public/ViewPublication/812781). For instance, the National Occupant Protection Use Survey Controlled Intersection Study uses a probability-based sample of observational surveys conducted on an annual basis to produce estimates of seat belt use nationwide at a typical daylight moment. Results during 2016–2018 indicate that seat belt use among adolescents and young adults aged 16–24 years was approximately 87% each year, whereas seat belt use among adults aged ≥25 years was 90% or higher (https://crashstats.nhtsa.dot.gov/Api/Public/ViewPublication/812781). Previous research also demonstrates that high school students put themselves at risk by riding with drivers who have been drinking alcohol (*2*).

Per mile driven, drivers aged 16–19 years have crash rates approximately four times greater than those of drivers aged ≥20 years (*1*); a leading contributor is driver inexperience (*1*,*3*). Because of this elevated crash risk, engagement in driver-related transportation risk behaviors (e.g., driving after drinking alcohol or texting or e-mailing while driving) puts adolescents at even higher risk. For example, drinking alcohol negatively affects a person’s ability to drive safely regardless of age. However, even at the same blood alcohol concentration (BAC), drivers aged 16–20 years have a much higher risk for being involved in a crash than older drivers (*1*,*4*). Similarly, the negative effects of driver inexperience on driving performance are worsened by cell phone–related driver distraction (*5*).

For this report, 2019 data from the Youth Risk Behavior Survey (YRBS) were analyzed by student characteristics to determine the prevalence of four transportation risk behaviors among U.S. high school students. Associations between engagment in multiple transportation risk behaviors also were calculated. This study provides an update on which adolescent groups have an elevated prevalence of engaging in transportation risk behaviors and reveals the extent to which adolescents engage in multiple transportation risk behaviors. The findings can help traffic safety and public health professionals appropriately select, tailor, and implement effective strategies to have a greater impact on reducing risk behaviors, thereby preventing crashes, injuries, and deaths among adolescents.

## Methods

### Data Source

This report includes data from CDC’s 2019 YRBS, a cross-sectional, school-based survey conducted biennially since 1991. Each survey year, CDC collects data from a nationally representative sample of public and private school students in grades 9–12 in the 50 U.S. states and the District of Columbia. Additional information about YRBS sampling, data collection, response rates, and processing is available in the overview report of this supplement (*6*). The prevalence estimates for all unintentional injury questions for the overall study population and by sex, race/ethnicity, grade, and sexual orientation are available at https://nccd.cdc.gov/youthonline/App/Default.aspx. The full YRBS questionnaire is available at https://www.cdc.gov/healthyyouth/data/yrbs/pdf/2019/2019_YRBS-National-HS-Questionnaire.pdf.

### Measures

This study examined two passenger- and two driver-related transportation risk behaviors among U.S. high school students. The overall analytic sample was used for the passenger-related risk behaviors, which included not always wearing a seat belt when riding in a car driven by someone else and riding with a driver who had been drinking alcohol (riding with a drinking driver). Not always wearing a seat belt was assessed with the question, “How often do you wear a seat belt when riding in a car driven by someone else?” Response options included “always,” “most of the time,” “sometimes,” “rarely,” or “never,” with any response other than “always” being defined as not always wearing a seat belt. Riding with a drinking driver was assessed with the question, “During the past 30 days, how many times did you ride in a car or other vehicle driven by someone who had been drinking alcohol?” Responses were dichotomized (0 times versus ≥1 time). Students who reported riding with a drinking driver at least once during the previous 30 days were classified as having engaged in the behavior.

Driver-related transportation risk behaviors included driving when they had been drinking alcohol (driving after drinking alcohol) and texting or e-mailing while driving (texting while driving). Driving after drinking alcohol was assessed with the question, “During the past 30 days, how many times did you drive a car or other vehicle when you had been drinking alcohol?” Texting while driving was assessed with the question, “During the past 30 days, on how many days did you text or e-mail while driving a car or other vehicle?” Students who indicated they had not driven a car or other vehicle during the past 30 days on each respective question were excluded from the analysis for these questions. Responses among drivers were categorized as 0 times or days versus ≥1 time or day.

An approximation of driving prevalence among students is presented to provide context for the driver-related behaviors. However, driving prevalence is not directly captured in the 2019 YRBS. For this approximation, students who chose a response other than “I did not drive a car or other vehicle during the past 30 days” for both driver-related questions (driving after drinking alcohol and texting while driving) were classified as drivers, and students who indicated that they did not drive a car or other vehicle during the past 30 days were classified as nondrivers. Driver classification was independent of students’ responses to the two questions about passenger-related transportation risk behaviors because students who drove during the past 30 days could also be passengers when they were not driving during the same 30-day period.

All transportation risk behaviors were analyzed by self-reported student characteristics, including age (14, 15, 16, 17, or ≥18 years), sex (male or female), race/ethnicity (non-Hispanic white [white]; non-Hispanic black [black]; or Hispanic or Latino of any race [Hispanic]), academic grades in school (mostly As or Bs versus mostly Cs, Ds, or Fs), and sexual identity (heterosexual; lesbian, gay, or bisexual; or not sure). Although data from students in other or multiple racial/ethnic groups were collected, the numbers were too small to produce statistically stable estimates specific to other or multiple racial/ethnic groups; therefore, these data are not presented as a separate group in this report but were retained in the analytic sample. In addition, students aged <14 years (n = 87) were not included in the analysis by age because the sample of students in this age category was too small for meaningful analysis and because these students cannot legally drive anywhere in the United States (*1*).

### Analysis

For this report, unadjusted weighted prevalence estimates and corresponding 95% confidence intervals were calculated, and posthoc *t*-tests were used to assess between-group differences. Differences between prevalence estimates were considered statistically significant if the *t*-test p value was <0.05. In the results, only statistically significant differences in prevalence estimates are reported.

Logistic regression models that controlled for age, sex, race/ethnicity, academic grades in school, and sexual identity produced adjusted prevalence ratios and examined the associations between transportation risk behaviors. For passenger-related transportation risk behaviors, students who did not engage in the risk behaviors were designated as the referent group. For driver-related transportation risk behaviors, students who drove but did not engage in the risk behaviors were designated as the referent group. Adjusted prevalence ratios were considered statistically significant if their pairwise comparison between groups (risk versus referent) was p <0.05.

## Results

In 2019, a total of 43.1% of U.S. high school students had not always worn a seat belt and 16.7% had ridden with a drinking driver during the 30 days before the survey (Table 1). Among the 59.9% of respondents who had driven a car or other vehicle during the 30 days before the survey, 5.4% had driven after drinking alcohol, and 39.0% had texted while driving.

**TABLE 1:** Unweighted number and unadjusted weighted prevalence estimates of high school students* who engaged in transportation risk behaviors, by selected characteristics — Youth Risk Behavior Survey, United States, 2019

Characteristic	Total	% (95% CI)	Did not always wear a seat belt^¶^		Rode with a driver who had been drinking alcohol**		Drove when they had been drinking alcohol**^,††^		Texted or e-mailed while driving^††,§§^	
	No.^†^		No.^§^	% (95% CI)	No.^§^	% (95% CI)	No.^§^	% (95% CI)	No.^§^	% (95% CI)
**Total**	**13,677**	**NA**	**4,852**	**43.1 (40.2–45.9)**	**2,214**	**16.7 (15.2–18.2)**	**423**	**5.4 (4.5–6.5)**	**2,784**	**39.0 (36.4–41.7)**
**Age (yrs)^¶¶^**										
14	**1,699**	** 11.9 (10.9–13.0)**	573	45.7 (40.9–50.5)	276	16.4 (13.9–19.1)	14	2.7 (0.9–7.5)	51	15.5 (11.2–21.0)
15	**3,473**	** 24.8 (23.5–26.0)**	1,283	46.9 (42.7–51.1)	557	16.7 (14.6–19.1)	49	2.6 (1.7–3.9)	211	15.5 (11.8–20.2)
16	**3,628**	**25.6 (24.5–26.7)**	1,318	43.5 (39.5–47.6)	564	16.0 (13.8–18.5)	112	4.0 (2.8–5.6)	730	30.5 (25.8–35.5)
17	**3,102**	**23.7 (22.5–24.8)**	1,045	38.9 (35.6–42.4)	481	16.0 (13.8–18.5)	138	5.9 (4.3–7.9)	1,072	50.9 (46.5–55.3)
≥18	**1,616**	**13.7 (12.6–14.9)**	574	39.4 (36.6–42.4)	279	18.4 (15.4–21.7)	91	8.9 (6.4–12.4)	672	59.5 (54.9–63.9)
**Sex**										
Male	**6,641**	50.6 (49.1–52.1)	2,369	43.3 (40.0–46.7)	1,015	15.6 (14.1–17.2)	257	7.0 (5.6–8.8)	1,434	39.6 (36.6–42.6)
Female	**6,885**	49.4 (47.9–50.9)	2,440	42.7 (39.7–45.7)	1,141	17.5 (15.6–19.5)	149	3.6 (2.8–4.6)	1,311	38.4 (35.5–41.4)
Race/Ethnicity***										
White, non-Hispanic	**6,668**	51.2 (46.4–56.0)	2,079	36.6 (33.8–39.6)	986	15.1 (13.5–16.8)	207	5.1 (3.9–6.5)	1,608	43.9 (40.4–47.5)
Black, non-Hispanic	**2,040**	12.2 (10.2–14.6)	901	61.7 (56.3–66.8)	325	15.9 (13.3–18.7)	47	4.1 (2.6–6.4)	312	29.5 (24.3–35.2)
Hispanic	**3,038**	26.1 (21.8–30.9)	1,237	48.2 (45.0–51.4)	605	20.8 (18.7–23.1)	107	6.6 (5.2–8.5)	562	35.2 (30.8–39.8)
**Academic grades** ^†††^										
Mostly As or Bs	**9,785**	75.1 (72.2–77.8)	3,152	38.8 (36.0–41.6)	1,449	15.3 (13.8–17.0)	248	4.7 (3.8–5.9)	2,070	40.4 (37.8–43.1)
Mostly Cs, Ds, or Fs	**2,677**	20.6 (18.3–23.2)	1,226	57.0 (53.4–60.5)	547	20.1 (17.7–22.8)	133	7.4 (5.7–9.6)	548	37.1 (32.2–42.4)
**Sexual identity**										
Heterosexual	**10,853**	84.4 (83.4–85.3)	3,741	42.1 (39.1–45.2)	1,656	15.7 (14.1–17.4)	322	5.2 (4.2–6.4)	2,268	39.6 (36.6–42.6)
Lesbian, gay, or bisexual	**1,531**	11.2 (10.4–12.0)	564	44.7 (39.4–50.1)	283	19.2 (16.0–22.9)	39	4.7 (2.4–9.0)	257	34.7 (28.4–41.7)
Not sure	**591**	4.5 (3.9–5.0)	208	43.3 (37.6–49.2)	125	21.9 (16.8–28.1)	24	9.5 (4.8–17.7)	93	31.7 (22.0–43.4)

**Abbreviations:** CI = confidence interval; NA = not applicable.

* Unadjusted weighted prevalence estimates and corresponding 95% CIs were calculated and are presented in the table. Posthoc *t*-tests were used to assess between-group differences. Differences were considered statistically significant if the *t*-test p value was <0.05. Statistical significance is not indicated in the table due to the large number of different pairwise comparisons; however, all significant differences are described in the results.

^†^ The unweighted number of students for each characteristic only includes students who selected a response on the survey question pertaining to that characteristic. Students who did not select a response were not included in the analysis for that characteristic but were retained in the analytic sample for every question on which they provided a response.

^§^ Students who selected any response on the survey question pertaining to a risk behavior were included in the analysis for that behavior; however, only the unweighted numbers of students who engaged in that behavior are presented in the table. Students who did not select a response were not included in the analysis for that behavior but were retained in the analytic sample for every question on which they provided a response.

^¶^ Most of the time, sometimes, rarely, or never wore a seat belt when riding in a car driven by someone else.

** ≥1 time during the 30 days before the survey.

^††^ Among students who had driven a car or other vehicle during the 30 days before the survey.

^§§^ On ≥1 day during the 30 days before the survey.

^¶¶^ The total column percentages for age do not add up to 100% because students aged <14 years are not presented because they cannot drive legally in any U.S. state.

*** The total column percentages for race/ethnicity do not add up to 100% because other non-Hispanic race categories are not presented.

^†††^ The total column percentages for academic grades do not add up to 100% because students who were not sure about their grades or who responded “none of these grades” are not presented.

Both driving after drinking alcohol and texting while driving usually increased with age. Specifically, prevalence of driving after drinking alcohol was higher among students aged ≥18 years (8.9%) than among students aged 16 (4.0%), 15 (2.6%), or 14 (2.7%) years (Table 1). In addition, prevalence was higher among students aged 17 (5.9%) years than among those aged 15 (2.6%) years. For texting while driving, prevalence was higher among students aged ≥18 (59.5%) years than among students aged 17 (50.9%), 16 (30.5%), 15 (15.5%), or 14 (15.5%) years. Prevalence also was higher among students aged 17 years than among those aged 16, 15, or 14 years and higher among students aged 16 years than among those aged 15 or 14 years.

Conversely, not always wearing a seat belt usually decreased with age. Prevalence of not always wearing a seat belt was lower among students aged ≥18 years (39.4%) than among students aged 16 (43.5%), 15 (46.9%), or 14 (45.7%) years. Similarly, prevalence was lower among students aged 17 years (38.9%) than among all younger students. For riding with a drinking driver, no differences occurred by age.

Differences by race/ethnicity were detected for all four transportation risk behaviors but did not demonstrate a consistent pattern. Prevalence of not always wearing a seat belt was higher among black students (61.7%) than among Hispanic students (48.2%) or white students (36.6%). In addition, prevalence among Hispanic students was higher than among white students. For the alcohol-related transportation risk behaviors, Hispanic students (20.8%) had a higher prevalence of riding with a drinking driver than black students (15.9%) or white students (15.1%), and Hispanic students (6.6%) had a higher prevalence of driving after drinking alcohol than black students (4.1%). In contrast, prevalence of texting while driving was higher among white students (43.9%) than among black students (29.5%) or Hispanic students (35.2%). Students whose academic grades in school were mostly Cs, Ds, or Fs had a higher prevalence of not always wearing a seat belt (57.0%), riding with a drinking driver (20.1%), and driving after drinking alcohol (7.4%) than students whose academic grades in school were mostly As or Bs (38.8%, 15.3%, and 4.7%, respectively); however, prevalence of texting while driving did not differ by this characteristic.

Few differences were identified when examining behaviors by sex and by sexual identity. Only alcohol-related transportation risk behaviors demonstrated differences. Among students who had driven during the 30 days before the survey, male students (7.0%) had a higher prevalence of driving after drinking alcohol than female students (3.6%). By sexual identity, students who were not sure of their sexual identity (21.9%) had a higher prevalence of riding with a drinking driver than heterosexual students (15.7%); however, the prevalence was not different from lesbian, gay, or bisexual students (19.2%).

Multivariable analyses indicated that, for each transportation risk behavior, students engaging in that behavior were more likely to engage in each of the other transportation risk behaviors, after controlling for age, sex, race/ethnicity, academic grades in school, and sexual identity (Table 2). For passenger-related transportation risk behaviors, students who did not always worn a seat belt were 1.80 times as likely to have ridden with a drinking driver, 2.73 times as likely to have driven after drinking alcohol, and 1.29 times as likely to have texted while driving than students who always wore a seat belt. Students who had ridden with a drinking driver during the 30 days before the survey were 1.42 times as likely to not always wear a seat belt, 9.87 times as likely to have driven after drinking alcohol, and 1.50 times as likely to have texted while driving than students who had not ridden with a drinking driver. For driver-related transportation risk behaviors, students who had driven after drinking alcohol at least once during the 30 days before the survey were 1.65 times as likely to not always wear a seat belt, 4.91 times as likely to have ridden with a drinking driver, and 2.38 times as likely to have texted while driving than students who had not driven after drinking alcohol. Students who had texted while driving on at least one day during the 30 days before the survey were 1.32 times as likely to not always wear a seat belt, 1.96 times as likely to have ridden with a drinking driver, and 12.64* times as likely to have driven after drinking alcohol than students who had not texted while driving.

**TABLE 2:** Adjusted prevalence ratios* for high school students who engaged in multiple transportation risk behaviors — Youth Risk Behavior Survey, United States, 2019

Transportation risk behavior^†^	Did not always wear a seat belt^§^	Rode with a driver who had been drinking alcohol^¶^	Drove when they had been drinking alcohol^¶,^**	Texted or e-mailed while driving**^,††^
	aPR (95% CI)	aPR (95% CI)	aPR (95% CI)	aPR (95% CI)
Did not always wear a seat belt^§^	NA	1.80 (1.59–2.04)	2.73 (1.81–4.11)	1.29 (1.19–1.41)
Rode with a driver who had been drinking alcohol^¶^	1.42 (1.32–1.53)	NA	9.87 (7.14–13.64)	1.50 (1.37–1.65)
Drove when they had been drinking alcohol^¶,^**	1.65 (1.40–1.95)	4.91 (4.17–5.77)	NA	2.38 (2.15–2.63)
Texted or e-mailed while driving**^,††^	1.32 (1.20–1.44)	1.96 (1.69–2.27)	12.64 (8.45–18.91)^§§^	NA

**Abbreviations:** aPR = adjusted prevalence ratio; CI = confidence interval; NA = not applicable.

* Multivariable logistic regression models that controlled for age, sex, race/ethnicity, academic grades, and sexual identity were used to produce the aPRs and corresponding 95% CIs presented in the table. The aPRs were considered statistically significant if the p value of their pairwise comparison between groups (risk versus referent) was <0.05. All aPRs in the table are significant.

^†^ Students who engaged in protective behaviors (i.e., always wearing a seat belt) or did not engage in risk behaviors (i.e., riding with a driver who had been drinking alcohol, driving when they had been drinking alcohol among students who had driven, or texting or e-mailing while driving among students who had driven) were the referent group.

^§^ Most of the time, sometimes, rarely, or never wore a seat belt when riding in a car driven by someone else.

^¶^ ≥1 time during the 30 days before the survey.

** Among students who had driven a car or other vehicle during the 30 days before the survey.

^††^ On ≥1 day during the 30 days before the survey.

^§§^ Estimate should be interpreted with caution because the 95% CI is wide.

## Discussion

Transportation risk behaviors varied by student characteristics, with age, race/ethnicity, and academic grades demonstrating the most differences. Increased engagement in driver-related transportation risk behaviors as students become older has been reported in other studies (*7*–*9*). This finding is not surprising because adolescents engage in certain risky driver–related behaviors less often when an adult supervisor is present in the vehicle, as is required when adolescents possess a driver’s permit (https://aaafoundation.org/distracted-driving-among-newly-licensed-teen-drivers/). As adolescents age, begin to drive without adult supervision, and gain driving experience, driver-related risk behaviors can be more common (*9*) (https://aaafoundation.org/distracted-driving-among-newly-licensed-teen-drivers). The positive association between age and texting while driving illustrates the need to sustain attention to preventing the behavior throughout adolescence (*9*). On the other hand, the prevalence of not always wearing a seat belt decreased by age, possibly indicating that although adolescents are typically more willing to engage in risky transportation behaviors as they become older, they still maintain a sense of self-preservation and risk perception and therefore take precautions by wearing seat belts.

This study demonstrated that Hispanic students had a higher prevalence of riding with a drinking driver and driving after drinking alcohol than white students or black students. One study described similar findings about drinking and driving among Hispanics in the literature (*10*). Additional research to explore which Hispanic populations might be at higher risk found that U.S.-born Hispanic youths were more likely to initiate drinking and driving behavior compared with first-generation immigrant Hispanic youths, even after adjusting for demographic variables (*10*). Additional research is needed to determine whether different strategies to reduce alcohol-impaired driving should be selected for or tailored to specific Hispanic populations based on nativity status.

Other studies have reported that students with lower academic grades were more likely to engage in other health-related risk behaviors (e.g., risky sexual behaviors or substance use) (*11*). The 2019 YRBS illustrates that this association extends to engagement in transportation risk behaviors. Lower academic achievement might be indicative of an underlying tendency to make riskier decisions, or risky behaviors themselves might lead to lower academic achievement. More research into a potential causal association and the temporality of that association is warranted. Of note, texting while driving was the one transportation risk behavior that did not differ by academic achievement. One potential explanation is that although adolescents understand that texting while driving is unsafe, the perceived benefits of texting while driving and the motivations for engaging in the behavior often differ from other transportation risk behaviors and can outweigh the perceived risks for adolescents at the moment when they choose to do it (*8*,*9*).

In this study, students engaging in any given transportation risk behavior were more likely to engage in each of the other measured transportation risk behaviors, even after controlling for student characteristics. Associations with alcohol-related behaviors were highest, particularly for driving after drinking alcohol. Students who engaged in any of the other transportation risk behaviors were approximately 3–13 times as likely to have also engaged in driving after drinking alcohol at least once during the 30 days before the survey. This might signify a general willingness to engage in risky behaviors among students who choose to drink and drive. This finding is also concerning because of the potential additive effects of these transportation risk behaviors. For example, adolescents who drive after drinking alcohol, thus increasing their risk for a crash, are also more likely to not always wear a seat belt, which increases their risk for injury or death during a crash.

Because students engaged in multiple transportation risk behaviors, interventions designed to address multiple transportation risk behaviors might concurrently help reduce those behaviors. Existing infrastructure and resources for comprehensive school and community programs designed to address different health behaviors could be leveraged to expand the benefits of these programs to transportation risk behaviors. For example, programs that already rely on family engagement could incorporate safe driving, because parental involvement is crucial for teaching adolescents how to drive by providing varied practice opportunities, promulgating safe driver behaviors, and instilling the importance of avoiding transportation risk behaviors (https://www.cdc.gov/parentsarethekey/parents/index.html). Programs that provide counseling and social services for adolescents could incorporate brief alcohol interventions, which are promising for reducing drinking and driving among adolescents at high risk for engaging in the behavior (*12*,*13*).

Engagement in all of these transportation risk behaviors across the United States remains high. Considering that adolescent drivers (16–19 years of age) have the highest crash rates (*1*), the fact that only six of 10 adolescents in this study always wore seat belts is concerning. Measures that are effective for increasing seat belt use, such as primary enforcement seat belt laws that allow police to ticket drivers or passengers for being unrestrained even in the absence of other violations (*13*), also can be beneficial for preventing crashes or crash injuries involving other contributing factors. For example, evidence indicates that primary enforcement seat belt laws are effective for reducing fatal alcohol-related crashes among underage drivers aged 15–20 years (*14*).

Although this study did not find many differences in riding with a drinking driver by student characteristics, approximately one of every five students engaged in the behavior. Riding with a drinking driver is intrinsically unsafe and also is associated with adolescent drinking and driving (*15*). Longitudinal research has revealed that adolescent passengers who are exposed to drinking and driving at a young age are more likely to engage in drinking and driving themselves as they become older and begin to drive (*16*). Additional research about the drinking drivers with whom adolescents ride and their relationships with the drinking drivers (e.g., parents, other family members, or peers) might be useful for designing and implementing targeted interventions.

In every U.S. state, minimum legal drinking age (MLDA) laws stipulate that drinking alcohol is illegal for anyone aged <21 years, as is driving after drinking any amount alcohol (zero tolerance laws) (*1*,*13*). Despite these laws, approximately one fifth of drivers aged 16–20 years killed in crashes during 2018 had blood alcohol concentrations (BACs) of ≥0.08% (*1*). This study found that in 2019, a total of 5.4% of students who drove did so after drinking alcohol at least once in the previous 30 days. Driving after drinking alcohol is risky and unacceptable at any age; however, the risk is even higher among adolescent drivers 16–20 years of age, even at BACs below the legal limit for adults (*4*). Zero tolerance laws (*7*,*13*,*14*), graduated driver licensing systems (*7*), and MLDA laws (*7*,*13*,*14*) are effective in helping reduce drinking and driving and alcohol-related crashes and injuries among adolescents, and they should continue to remain universally implemented. Other general population deterrent approaches that are effective for preventing alcohol-impaired driving overall also can be beneficial for specifically preventing adolescent drinking and driving. For example, publicized sobriety checkpoints are highly effective for reducing drinking and driving overall (*13*), and evidence indicates that they can reduce alcohol-impaired driving (*17*) and alcohol-related crashes among underage drivers (*14*).

Consistent with two recent studies, this analysis determined that texting while driving among adolescents remains high, increases with age, and is more common among white students than students of other races/ethnicities (*8*,*9*). Similar to the other studies, this analysis also determined that adolescents who engage in texting while driving are more likely to engage in other transportation risk behaviors (*8*,*9*). Awareness campaigns, education, and changes in policy related to texting while driving have had mixed effectiveness (*9*,*13*). Because of this, such technologic interventions as in-vehicle cell phone blocking technologies can serve as potential solutions; however, the effectiveness and acceptability of such solutions require more research (*9*,*13*).

Lack of parental monitoring and supervision is a common underlying contributor to many health risk behaviors, and parental involvement can be especially important for reducing transportation risk behaviors. For example, one study found that adolescents with supportive parents who monitor their behavior were less likely to engage in multiple passenger- and driver-related transportation risk behaviors, including seat belt nonuse, cell phone use while driving, and drinking and driving, than adolescents with uninvolved parents (*18*). Parents/guardians also can play a vital role in teaching adolescents to drive by helping ensure they gain valuable driving experience and by setting rules and expectations for adolescent drivers, including rules and expectations for not engaging in transportation risk behaviors. Parent-teen driving agreements (https://www.cdc.gov/parentsarethekey/parents/index.html) can formalize those expectations and demonstrate a commitment between parents and adolescents to adhere to safe driving practices while adolescents gain new driving privileges over time.

## Limitations

General limitations for the YRBS are available in the overview report of this supplement (*6*). The findings in this report is subject to at least two additional limitations. First, YRBS does not quantify driving or riding exposure in general or during the 30 days before the survey. How many trips each student takes as a driver or as a passenger and the amount of time each student spends on the road are unknown. High school students who take more frequent trips or drive for longer times or distances might have more opportunity to engage in transportation risk behaviors because of a higher exposure that is not captured by the survey. Second, for riding with a driver who had been drinking alcohol, the relationship between the student and the drinking driver (e.g., parent/guardian, other family member, a peer, or someone else) is unknown. The nature of this relationship might have implications for designing potential strategies and prevention messages for empowering adolescents so that they can intervene (*15*).

## Conclusion

Motor-vehicle–crash injuries remain a leading cause of death among adolescents. Despite this, passenger- and driver-related transportation risk behaviors that increase the risk for crashes, injuries, and deaths remain too common. Reducing transportation risk behaviors among adolescents by using proven strategies, especially those that can target multiple transportation risk behaviors, can help prevent crashes, reduce injuries, and save lives. Because driver-related transportation risk behaviors increased with age, continued emphasis on implementation of effective strategies for preventing these behaviors with high school juniors and seniors should be considered.

## Footnotes

* Estimate should be interpreted with caution because the 95% confidence interval is wide.
